# Clinical Profiles, Laboratory Biomarkers, and Mortality in Cancer Patients with Lower Respiratory Tract Infections: A Prospective Cohort Study

**DOI:** 10.3390/medicina60060901

**Published:** 2024-05-29

**Authors:** Samah Radwan, Dalia F. Mourad, Rana Hamdy, Mahmoud M. Kamel, Ahmed S. Abdel-Moneim, Dina M. Elkhashab, Dalia Y. Kadry

**Affiliations:** 1 Clinical Pathology Department, National Cancer Institute, Cairo University, Cairo 12613, Egypt; 2 Pediatric Oncology Department, National Cancer Institute, Cairo University, Cairo 12613, Egypt; 3 Department of Microbiology, College of Medicine, Taif University, Al-Taif 21974, Saudi Arabia

**Keywords:** lower respiratory tract infection, cancer, biomarkers, hematology, viral infections, bacterial infections, viruses, bacteria

## Abstract

Respiratory tract infections (RTIs) pose a substantial health burden worldwide, especially among immunocompromised groups like cancer patients. The aim of this prospective cohort study was to explore lower respiratory tract infections in cancer patients. We followed 107 cases with clinically or radiologically suspected lower respiratory tract infections until discharge or death, comprising 65 males and 42 females across diverse age groups. Clinical evaluations, including patient history, examination, and malignancy diagnosis, were conducted. Nasopharyngeal swabs (NPSs), sputum samples, and blood samples were collected within 24 h of symptom onset. Multiplex Real-Time PCR allowed for the simultaneous detection of viral, bacterial, and fungal infections, while conventional microbiological culture methods were used for bacterial and fungal analysis. SARS-CoV-2 infection was excluded in all of the enrolled patients using real-time RT-PCR. Hematological and biochemical analyses included hemoglobin, lymphocyte, neutrophil, and platelet counts, along with ALT, AST, creatinine, and CRP levels. Significant differences were noted in clinical presentations, management outcomes, and prognostic markers among patients with different hematological malignancies. Distinct clinical profiles were identified for leukemia, lymphoma, and solid tumors, with variations in age distribution and symptom prevalence. ICU admission rates varied significantly, with solid tumor patients exhibiting higher rates. The hematological and biochemical biomarkers differed across malignancies, with notable associations between lymphopenia, thrombocytopenia, and mortality following respiratory episodes. This study highlights the critical role of rapid pathogen detection and infection control measures in safeguarding vulnerable cancer patients from nosocomial transmission.

## 1. Introduction

Respiratory tract infections (RTIs) represent a significant health burden globally, particularly among immunocompromised populations such as cancer patients. Lower respiratory tract infections (LRTIs), including pneumonia and bronchitis, pose serious challenges in management due to their potential for severe complications and adverse outcomes. Cancer patients, with their compromised immune systems and susceptibility to infections, are particularly vulnerable to LRTIs. The immunosuppressive effects of cancer treatments further exacerbate this vulnerability, making early diagnosis and prompt management crucial for improving patient outcomes [1,2,3,4].

Cancer patients often present with nonspecific respiratory symptoms, including fever, cough, dyspnea, and chest pain. However, distinguishing between respiratory tract infections and cancer progression or treatment-related complications can be challenging. This highlights the importance of comprehensive clinical assessment and microbiological investigations to identify the underlying etiology and guide appropriate treatment strategies [5,6,7].

Various pathogens, including bacteria, viruses, fungi, and opportunistic microorganisms, can cause LRTIs in cancer patients. Common bacterial pathogens implicated in pneumonia include *Streptococcus pneumoniae*, *Haemophilus influenzae*, and *Staphylococcus aureus*. Viral respiratory pathogens, such as influenza viruses, respiratory syncytial virus (RSV), and adenovirus, are also significant contributors to LRTIs, particularly in immunocompromised individuals. Moreover, opportunistic fungal infections, such as invasive aspergillosis and *Pneumocystis jirovecii pneumonia* (PCP), pose a considerable risk to cancer patients undergoing chemotherapy or stem cell transplantation [8,9,10,11].

Despite advancements in diagnostic techniques, challenges remain in accurately identifying the causative pathogens of LRTIs in cancer patients. Molecular methods, including polymerase chain reaction (PCR) assays, offer sensitive and specific detection of viral and atypical bacterial pathogens. However, conventional microbiological cultures are still necessary for identifying antibiotic-resistant bacteria and guiding antimicrobial therapy. Additionally, the interpretation of microbiological results in cancer patients can be complex due to the presence of colonization, environmental contamination (e.g., from hospital surfaces, medical equipment, and healthcare personnel), bacterial biofilms, and previous antimicrobial exposure [12,13].

The clinical management of LRTIs in cancer patients involves a multidisciplinary approach, including antimicrobial therapy, supportive care, and, in severe cases, intensive care unit (ICU) admission and mechanical ventilation. The early initiation of empirical antibiotics is essential for reducing mortality in cancer patients with suspected bacterial pneumonia. However, antimicrobial stewardship is critical to prevent the emergence of multidrug-resistant pathogens and opportunistic infections. Furthermore, supportive measures, such as supplemental oxygen therapy, bronchodilators, and chest physiotherapy, play a vital role in alleviating respiratory symptoms and preventing complications [14,15].

Despite significant progress in understanding the epidemiology and clinical management of LRTIs in cancer patients, several gaps exist in our knowledge. These include the optimal diagnostic approach for identifying respiratory pathogens, the role of novel biomarkers in predicting disease severity and treatment response, and the impact of emerging infectious threats, such as COVID-19, on cancer patients’ outcomes. Addressing these gaps requires collaborative research efforts, incorporating clinical data, microbiological findings, and immunological parameters to tailor interventions to the unique needs of cancer patients with respiratory infections.

The aim of the present prospective cohort study was to investigate the clinical characteristics, microbiological etiology, and management outcomes of lower respiratory tract infections in cancer patients. Specifically, the authors sought to characterize the clinical presentation and disease severity of LRTIs in cancer patients and evaluate the utility of hematological and biochemical biomarkers in predicting disease progression and clinical outcomes in cancer patients with LRTIs.

## 2. Materials and Methods

### 2.1. Sample Collection and Processing

This study obtained approval from the Institutional Review Board of Cairo University’s National Cancer Institute (Approval No. 2207-310-031, dated 22 July 2022). This prospective cohort investigation screened all hospitalized individuals from the medical oncology and pediatric oncology departments at the National Cancer Institute, Cairo University, from 1 April 2023 to 31 August 2023, for lower respiratory tract infections. A total of 107 cancer patients, suspected clinically or radiologically of lower respiratory tract infection, were observed until discharge or death. The cohort consisted of 65 males and 42 females, spanning ages from under 1 year to over 60 years old. The cancer patients included 75 initial cancer cases, and 32 were follow-up cancer cases. Each participant underwent a thorough clinical evaluation, encompassing medical history, clinical examination, and complete diagnosis to determine the type of malignancy. In addition to blood samples, nasopharyngeal swabs were collected, mainly from children, while both nasopharyngeal swabs and sputum samples were collected from adult patients, all within 24 h of symptom onset. All patients were initially screened and confirmed to be free from SARS-CoV-2 viral infection.

### 2.2. Molecular and Classical Detection of Respiratory Infections in Cancer Patients

Multiplex Real-Time PCR, the Fast Track FTD-33 Test Kit (Fast Track Diagnostics), was used to identify respiratory tract infections comprehensively. The VERSANT^®^ Sample Preparation 1.0 Reagents kit facilitated nucleic acid extraction, carried out via the automated APEX KING FISHER platform from Thermofisher Scientific, München, Germany. Genomic RNA isolated from respiratory specimens underwent reverse transcription using specific primers, followed by immediate polymerase chain reaction (PCR) within the same tube. Concurrently, PCR amplification occurred for various pathogens’ DNA within the same tube. All experiments were executed using the QuantStudio^TM^ 5 Real-Time PCR system. The panel comprised 12 bacterial targets, 20 viral targets, and 1 fungal target (*P. jirovecii*). Among the bacterial targets were *H. influenzae*, *Bordatella* species (excluding *B. parapertussis*), *M. catarrhalis*, *Salmonella* species, *L. pneumophila/longbeachiae*, *K. pneumoniae*, *S. aureus*, *S. pneumoniae*, *C. pneumoniae*, and *M. pneumoniae*. On the viral spectrum, the targets encompassed influenza A, B, C, including subtype H1N1*pdm09*, rhinovirus, coronaviruses (NL63, 229E, OC43, HKU1), parainfluenza viruses (PIV-1–4), metapneumoviruses (A and B), bocavirus, hRSV serotypes (A and B), human adenovirus, enterovirus, and parechovirus. For routine classical microbiological examination, a second sample (nasopharyngeal swab and/or sputum) underwent immediate processing using conventional culture methods for bacteria, with automated diagnosis conducted using Vitec 2. Additionally, fungal culture was performed on Sabouraud dextrose agar.

### 2.3. Hematological and Biochemical Analysis

Hematological parameters, including lymphocyte, neutrophil, and platelet counts, were assessed utilizing a standardized automated hematology analyzer (SYSMEX XN-9100^TM^ Sysmex Corporation, Kobe, Japan). The quantification of ALT, AST, and creatinine was measured using the relevant kits according to the manufacturer’s instructions (Beckman Coulter AU 680, Miami, FL, USA). CRP levels in the serum were determined using a high-sensitivity CRP assay kit (Catalog Number SE120041, Sigma-Aldrich Co., St. Louis, MO, USA). This specialized assay kit is designed to detect low concentrations of CRP.

### 2.4. Statistical Analysis

The investigation employed various statistical techniques, including ANOVA, Chi-square test, Kruskal–Wallis test, Mann–Whitney U test, and independent samples *t*-test. A multivariate analysis was conducted using the Generalized Linear Model (GLM) to analyze the mixed data, accommodating both non-parametric and parametric variables.

## 3. Results

### 3.1. Pathogens Detected in Cancer Patients with Lower Respiratory Tract Infections

Among the 107 examined patients, 48 showed bacterial infection with a single bacterial pathogen (mainly Coagulase-Negative *Staphylococci* and *Klebsiella pneumoniae*), 3 showed fungal infection (*Pneumocytis jirovecii* and *Candida albicans*), and 1 showed viral infection (H1N1*pdm09*). Mixed infections involving more than one pathogen were detected in 42 patients, including 16 with mixed bacterial and viral infections; 2 with mixed bacterial, viral, and fungal infections; 12 with mixed bacterial infections; and 13 with mixed bacterial and fungal infections. Meanwhile, pathogens were not detected in 11 patients using the adopted methods in the current study (Table 1). The mixed viral infection included rhinovirus, influenza B virus, influenza C virus, HCoV-229, HCoV-OC43, HCoV-KHU1, PIV-3, and PIV-4. Mixed bacterial infection included Staphylococcus aureus, *Haemophilus influenzae*, *Streptococcus pneumoniae*, *Legionella pneumonia*, and *Moraxella catarrhalis.*

### 3.2. The Clinical Profile and Management Outcomes of Patients with Different Hematological Malignancies

The average age differed significantly across various malignancies (*p* < 0.031). Individuals with solid tumors exhibited a notably higher mean age (41.42 years) compared to those with leukemia (28.30 years) and lymphoma (21.73 years). No notable variance was observed in the gender distribution among different malignancies. Various clinical indicators were examined, encompassing fever, rhinorrhea, dyspnea, tachypnea, dry cough, productive cough, crepitation, and wheezy chest. Statistically significant distinctions were noted in the prevalence of dyspnea (*p* < 0.062) and tachypnea (*p* < 0.016) among different malignancies. The percentage of patients requiring ICU admission significantly varied among malignancies (*p* < 0.033). However, there was no notable contrast in ICU stay duration across different malignancies. Considerably elevated rates of mechanical ventilation were observed among patients with solid tumors compared to other malignancies (*p* < 0.001). Nonetheless, the duration of ventilator support did not display significant differences among different malignancies (Table 2).

### 3.3. Comparative Analysis of Hematological and Blood Biochemical Biomarkers in Patients with Various Malignancies

Between 33 and 42% of patients with leukemia, lymphoma, and solid tumors died following an LRTI. Significant differences were observed in Hb levels among different malignancies (*p* < 0.001). The least significant difference (LSD) revealed significant variation between the Hb concentration in solid tumors (9.73 ± 1.72) and leukemia (8.46 ± 2.10) (*p* < 0.001). Similarly a significant difference was reported between lymphoma (9.43 ± 2.04) and leukemia (*p* < 0.05). No significant reference was detected between lymphoma and solid tumors. Highly significant variation was detected in the leukocytic count in leukemia patients in comparison to other malignancies (*p* < 0.001). The lymphocyte count also showed significant differences among malignancies (*p* < 0.001), with the highest prevalence of lymphopenia observed in patients with solid tumors (27/50, 64.3%) and the highest prevalence of lymphocytosis observed in leukemia patients (24/42, 48%). Similarly, significant differences were noted in the distribution of neutrophils among malignancies (*p* < 0.001), with patients with solid tumors showing the highest prevalence of neutrophilia (25/42, 59.5%), while the highest prevalence of lymphopenia was observed in leukemia patients (39/50, 78.0%). The platelet profile also varied significantly among malignancies (*p* < 0.001), with patients with leukemia having a higher prevalence of thrombocytopenia and those with lymphoma having a higher prevalence of thrombocytosis. Creatinine, ALT, AST, CRP, Pt, PC, and INR levels did not exhibit significant differences among malignancies (Table 3).

### 3.4. Impact of ICU Admission, Hematological, and Biochemical Biomarkers on Mortality Rate in Cancer Patients Following Respiratory Episodes

Following respiratory episodes that were mostly due to infections in cancer patients, 63 survived and 47 died. Notably, there was a significant difference in ICU admission between survivors and non-survivors (*p* = 0.001), with a higher proportion of non-survivors requiring ICU care. The duration of the ICU stay was also significantly longer for non-survivors compared to survivors (*p* < 0.001). Mechanical ventilation was more frequently required among non-survivors (*p* < 0.001), with a longer duration of ventilator support observed, although this difference was not statistically significant. Analyzing blood gas parameters, non-survivors exhibited lower pH levels compared to survivors (*p* < 0.006), along with higher pCO2 levels (*p* < 0.031). However, no significant differences were observed in pO2 levels between the two groups (Table 3).

There was a significant difference in hemoglobin (Hb) levels between survivors and non-survivors (*p* < 0.014). Thrombocytopenia was significantly more prevalent in non-survivors (*p* < 0.012), indicating a potential association with mortality. Furthermore, there were no significant differences in PT, PC, or INR between survivors and non-survivors. Neutrophil, CRP, creatinine ALT, AST, and LDH levels did not show any statistically significant differences between survivors and non-survivors (Table 4).

### 3.5. Multivariate Analysis of Clinical Variables and Patient Outcomes

The multivariate analysis reveals significant associations between various clinical variables and patient outcomes in the intensive care setting. The corrected model in MANOV indicates significant effects on several dependent variables, including creatinine, pH, pCO_2_, thrombocyte, hemoglobin (Hb), and total leukocyte count (TLC). Notably, age is significantly associated with creatinine (*p* = 0.038) and TLC (*p* = 0.041). The number of ICU stay days shows a significant impact on creatinine (*p* = 0.009) and thrombocyte (*p* = 0.025). Mechanical ventilation significantly affects C-reactive protein (CRP) (*p* = 0.049), alkalosis (*p* = 0.041), and pH (*p* = 0.029). Additionally, the type of tumor significantly influences the pH (*p* = 0.027), pCO2 (*p* = 0.005), neutrophil (*p* = 0.005), Hb (*p* = 0.015), TLC (*p* = 0.001), ALT (*p* = 0.012), and AST (*p* = 0.002) (Table 5).

The multivariate analysis of variance (MANOVA) revealed significant findings across several parameters. The corrected model significantly predicted neutrophil status (*p* < 0.017), hemoglobin levels (*p* < 0.023), total leukocyte count (TLC) (*p* < 0.005), and ICU stay days (*p* < 0.023). The intercepts for variables, such as leukocyte count, LDH, AST, creatinine, CRP, pH, and others, highlighted the significant contributions of baseline values. Furthermore, the analysis showed that ICU stay days significantly impacted fatality (*p* < 0.002). The type of tumor significantly influenced neutrophil counts (*p* < 0.002), hemoglobin levels (*p* < 0.020), TLC (*p* < 0.021), and leukocyte count (*p* < 0.021). Additionally, TLC influenced both fatality and the type of tumor (*p* < 0.034).

## 4. Discussion

This study offers a comprehensive understanding of how different types of hematological malignancies affect patients’ clinical profiles and management outcomes, revealing crucial insights that call for thorough examination. In our investigation, bacterial infection emerged as the most detected pathogen. Both staphylococcal species and *Klebsiella pneumoniae*, a well-recognized Gram-negative bacterium, have consistently been identified as significant pathogens in nosocomial infections and among cancer patients [16,17,18]. Their capacity to form biofilms and develop resistance to antibiotics adds further complexity to treatment strategies [19]. These findings are consistent with the existing literature, which highlights the heightened vulnerability of cancer patients to nosocomial infections, often attributed to their compromised immune systems and frequent exposure to healthcare environments [11,17,20].

Significant differences in the average age across different malignancies suggest demographic heterogeneity within hematological cancers. Solid tumors are linked with a notably higher mean age compared to leukemia and lymphoma, indicating potential variations in disease etiology and progression [21]. The absence of significant variance in gender distribution among different malignancies indicates that hematological cancers may affect individuals irrespective of gender. This finding aligns with prior research demonstrating comparable incidence rates among males and females for certain hematological malignancies [22,23].

The examination of various clinical indicators, such as fever, dyspnea, and cough, provides valuable insights into the symptomatology of hematological malignancies. Significant distinctions were observed in the prevalence of dyspnea and tachypnea among different malignancies, which were also reported in other previous studies [24,25]. There was significant variation in the percentage of patients requiring ICU admission across malignancies, highlighting diverse clinical trials and illness severity among hematological cancer patients. This finding emphasizes the need for tailored critical care approaches and resource allocation based on disease-specific factors and patient characteristics, as previously described [26,27]. 

Survival rates varied among different hematological malignancies, with leukemia, lymphoma, and solid tumors exhibiting low survival outcomes. This finding matched those reported in other studies and suggests that the type of cancer significantly influences patient prognosis [28,29]. 

Significant differences in hemoglobin (Hb) levels were observed across malignancies, with patients with leukemia showing the lowest mean Hb level, which agrees with other studies [30,31]. These findings assume variations in erythropoietin production, bone marrow function, and treatment-related factors such as chemotherapy-induced myelosuppression.

Variations in lymphocyte (LC) and neutrophil distribution highlight potential differences in immune responses among malignancies. The higher prevalence of lymphopenia in patients with solid tumors may indicate impaired immune function, while the higher prevalence of neutrophilia suggests an inflammatory response, characteristic of tumor microenvironments [32,33,34,35]. 

Platelet profiles varied significantly among malignancies, with patients with leukemia exhibiting a higher prevalence of thrombocytopenia and those with lymphoma showing a higher prevalence of thrombocytosis. These findings may reflect differences in platelet production, consumption, and the underlying pathophysiology of hematological cancers [36,37,38]. 

Creatinine levels exhibited significant differences among malignancies, with patients with solid tumors having the highest mean creatinine level. These variations imply differences in renal functions, tumor-related renal impairment, or nephrotoxic effects of chemotherapy [39,40,41]. 

The impact of ICU admission, hematological, and biochemical biomarkers on mortality rates in cancer patients following respiratory episodes reveals crucial insights into the complex interplay of clinical factors influencing patient outcomes. The significant difference in ICU admission rates between survivors and non-survivors highlights the critical role of intensive care in managing cancer patients experiencing respiratory episodes. The higher proportion of non-survivors requiring ICU care denotes the severity of respiratory complications in this subgroup, in agreement with other relevant studies [42,43,44,45,46]. 

Furthermore, the prolonged duration of ICU stay among non-survivors emphasizes the challenges associated with managing critically ill cancer patients, as well as the need for comprehensive and prolonged supportive care [47,48]. The longer duration of the ICU stay may also reflect the complexity of medical management and the presence of comorbidities that contribute to poorer prognosis in this patient population. 

Mechanical ventilation emerged as a crucial intervention in managing respiratory failure in cancer patients, with higher frequencies observed among non-survivors [49]. Elevated rates of mechanical ventilation among patients with solid tumors denote the increased risk of respiratory complications in this subgroup. While the duration of ventilator support did not significantly differ among malignancies in the current study, the need for mechanical ventilation points out the critical nature of respiratory management in hematological cancer patients [24,47,48].

The lower pH levels and higher pCO2 levels observed in non-survivors in the current study indicate the presence of respiratory acidosis and hypoventilation, reflecting the severity of respiratory compromise and the need for aggressive ventilatory support. This emphasizes the fact that analyzing blood gas parameters provides valuable insights into the physiological derangements associated with respiratory failure [50].

Hematological and biochemical biomarkers play a crucial role in predicting mortality risk and guiding clinical management in cancer patients with respiratory episodes [51,52,53]. While no significant differences were observed in hemoglobin levels between survivors and non-survivors, lower lymphocyte counts and higher prevalence of thrombocytopenia were associated with an increased mortality risk. These findings underscore the importance of immune dysregulation and hematological abnormalities in predicting outcomes in this patient population. Furthermore, elevated levels of CRP, a marker of systemic inflammation, were significantly associated with mortality in cancer patients following respiratory episodes [54]. This highlights the role of inflammatory processes in driving disease progression and influencing patient outcomes in cancer-related respiratory complications. The MANOVA of variables in the current study indicates that leukocyte counts, neutrophil status, and hemoglobin levels particularly showed the significant impact on the fatal consequence of LRTIs and varied significantly among different types of tumors.

## 5. Conclusions

In conclusion, our study offers a comprehensive examination of hematological malignancies’ and solid cancer patients’ impact on patients’ clinical profiles and outcomes post-respiratory episodes. We highlight demographic heterogeneity, clinical indicator significance, and survival rate disparities across malignancies. Our findings emphasize the importance of tailored critical care interventions and personalized treatment strategies based on the cancer subtype. The association between elevated CRP levels and mortality stresses the role of inflammatory processes in disease progression. 

## Figures and Tables

**Table 1:** Infection distribution and mortality rates among cancer patients with lower respiratory disease in intensive care unit.

Pathogen	Fatality	Type of Tumor			Cumulative
		Leukemia (N = 50)	Lymphoma (N = 15)	Solid Tumor (N = 42)	
Not known ^a^	Non-fatal	6	0	3	9
	Fatal	1	0	1	2
Single viral infection	Non-fatal	0	0	1	1
	Fatal	0	0	0	0
Single Bacterial infection	Non-fatal	16	2	10	28
	Fatal	10	3	7	20
Single fungal infection	Non-fatal	0	2	0	2
	Fatal	0	0	1	1
Mixed bacterial infections	Non-fatal	1	1	1	3
	Fatal	5	1	3	9
Mixed viral and bacterial infections	Non-fatal	2	1	5	8
	Fatal	3	1	3	7
Mixed viral, bacterial and fungal infections	Non-fatal	0	0	2	2
	Fatal	0	0	0	0
Mixed bacterial and fungal infections	Non-fatal	4	3	2	9
	Fatal	1	0	3	4

^a^ No known pathogen detected. Infections were screened using FTD-33 real-time PCR commercial kit and classical bacteriological screening of bacteria and fungi.

**Table 2:** Comparative analysis of the clinical profile and management outcomes of patients with different malignancies.

Item	Leukemia ^a^ N = 50	Lymphoma ^a^ N = 15	Solid Tumor N = 42	*p* Value
Age (years)	28.30 ± 19.60	21.73 ± 18.37	41.42 ± 25.44	0.031 *^b^
Sex				
Male	30 (60.0)	8 (53.3)	27 (64.3)	0.923 ^b^
Female	20 (40.0)	7 (46.7)	15 (35.7)	
Clinical Signs				
Fever	47 (88.7)	15 (100.0)	31 (79.5)	0.195 ^b^
Rhinorrhea	8 (15.1)	1 (6.7)	2 (5.1)	0.468 ^b^
Dyspnea	13 (24.5)	7 (46.7)	18 (46.2)	0.062 ^b^
Tachypnea	27 (50.9)	7 (46.7)	31 (79.5)	0.016 *^b^
Dry cough	28 (52.8)	10 (66.7)	20 (51.3)	0.748 ^b^
Productive cough	10 (18.9)	3 (20.0)	13 (33.3)	0.366 ^b^
Crepitation	12 (22.6)	3 (20.0)	11 (28.2)	0.852 ^b^
Wheezy chest	28 (52.8)	11 (73.3)	20 (51.3)	0.479 ^b^
ICU admission				
No	16 (32.0)	4 (26.7)	4 (9.5)	0.033 *^b^
Yes	34 (68.0)	11 (73.3)	38 (90.5)	
ICU stay (days)	5.97 ± 5.10	8.73 ± 8.79	7.74 ± 8.46	0.849 ^c^
Mechanical ventilation				
No	46 (92.0)	13 (86.7)	26 (61.9)	0.001 *^b^
Yes	4 (8.0)	2 (13.3)	16 (38.1)	
Ventilator days	6.75 ± 4.92	7.0 ± 4.24	8.76 ± 9.34	0.933 ^c^
Surgical removal				
No	50 (100)	15 (100.0)	3 (7.1)	0.001 *^b^
Yes	0 (0.0)	0 (0.0)	39 (92.9)	
Radiotherapy				
No	50 (100)	15 (100.0)	27 (64.3)	0.001 *^b^
Yes	0 (0.0)	0 (0.0)	15 (35.7)	
pH	7.41 ± 0.10	7.43 ± 0.06	7.38 ± 0.13	0.425 ^c^
pCO_2_	33.94 ± 8.42	34.45 ± 5.08	38.58 ± 10.77	0.209 ^c^
PO_2_	54.68 ± 33.39	46.32 ± 20.61	74.44 ± 45.50	0.076 ^c^
HCO_3_	21.89 ± 3.61	23.54 ± 3.71	22.83 ± 4.61	0.551 ^c^

^a^ Patients with acute lymphoblastic leukemia are treated with chemotherapy according to Jude Children’s Research Hospital (SJCRH) TOTXV protocol, while patients with NHL are treated according to LMB protocol. Both ^b^ Chi square and ^c^ ANOVA test were used for statistical analysis. The mean differences and Std. Deviation. * Significant at *p* < 0.05. ICU: Intensive Care Unit. HCO_3_: Bicarbonate. PO_2_: Partial Pressure of Oxygen. pCO_2_: Partial Pressure of Carbon Dioxide. pH: Potential of Hydrogen.

**Table 3:** Comparative analysis of hematological and blood biochemical biomarkers in patients with various malignancies.

	Leukemia N = 50	Lymphoma N = 15	Solid Tumor N = 42	*p* Value
Disease outcomes				
Alive	29 (58.0)	10 (66.7)	25 (59.5)	0.834 ^b^
Death	21 (42.0)	5 (33.3)	17 (40.5)	
Hb (g/dL) ^a^	8.46 ± 2.10	9.43 ± 2.04	9.73 ± 1.72	0.001 *^c^
LC	54. 4 ± 101.8	11.1 ± 7.9	10.9 ± 6.7	0.001 ^c^
Lymphocytes				
Lymphopenia	14 (28.0)	6 (40.0)	27 (64.3)	0.001 *^b^
Normal	12 (24.0)	9 (60.0)	10 (23.8)	
Lymphocytosis	24 (48.0)	0 (0.0)	5 (11.9)	
Neutrophils				
Neutropenia	39 (78.0)	4 (26.7)	5 (11.9)	0.001 *^b^
Normal	7 (14.0)	4 (26.7)	12 (28.6)	
Neutrophilia	4 (8.0)	7 (46.7)	25 (59.5)	
Platelet				
Thrombocytopenia	44 (88.0)	4 (26.7)	9 (21.4)	0.001 *^b^
Normal	6 (12.0)	6 (40.0)	28 (66.7)	
Thrombocytosis	0 (0.0)	5 (33.3)	5 (11.9)	
PT (seconds)	15.20 ± 3.35	14.20 ± 3.50	14.55 ± 2.56	0.522 ^c^
PC (%)	70.56 ± 18.36	79.03 ± 16.60	75.7 ± 15.19	0.233 ^c^
INR	1.27 ± 0.26	1.18 ± 0.24	1.21 ± 0.20	0.786 ^c^
ALT (U/L)	39.08 ± 73.51	23.73 ± 16.74	41.42 ± 53.34	0.593 ^c^
AST (U/L)	47.45 ± 52.12	33.00 ± 23.07	44.78 ± 42.64	0.683 ^c^
Creatinine (mg/dL)	0.78 ± 0.32	0.66 ± 0.26	0.93 ± 0.71	0.154 ^c^
CRP	136.99 ± 130.96	79.05 ± 94.59	134.3 ± 109.46	0.231 ^c^
LDH (U/L)	912.0 ± 1010.1	865.3 ± 506.1	605.8 ± 760.7	0.503 ^c^

^a^ The least significant difference (LSD) revealed significant variation between Hb concentration in solid tumor (9.73 ± 1.72) and leukemia (8.46 ± 2.10) (*p* < 0.001). Similarly significant difference was reported between lymphoma (9.43 ± 2.04) and leukemia (*p* < 0.05). Both ^b^ Chi square and ^c^ ANOVA test were used for statistical analysis. The mean differences and Std. Deviation, * Significant at *p* < 0.05. Hb: Hemoglobin. LC: Leukocyte count. PT: Prothrombin time. PC: Prothrombin concentration. INR: International normalized ratio. ALT: Alanine transaminase. AST: Aspartate transaminase. CRP: C-reactive protein. LDH: Lactate dehydrogenase.

**Table 4:** Clinical outcomes of cancer patients following lower respiratory tract infections (LRTIs).

Variable	Outcome of Infectious Episode		*p* Value
	Alive N = 64	Death N = 43	
ICU admission			
No	24 (38.1)	0 (0.0)	0.001 *^a^
Yes	40 (61.9)	43 (100.0)	
ICU stay (days)	4.6 ± 2.88	9.9± 9.4	0.001 *^b^
Mechanical ventilation			
No	58 (89.1)	27 (60.5)	0.001 *^a^
Yes	6 (10.9)	16 (39.5)	
Ventilator days	6.0 ± 4.52	8.78 ± 9.02	0.626 ^b^
pH	7.43 ± 0.07	7.36 ± 0.13	0.006 *^b^
pCo_2_	33.7 ± 6.75	38.5 ± 11.2	0.031 *^b^
pO_2_	63.5 ± 40.8	63.7 ± 39.8	0.501 ^b^
Hb (g/dL)	9.17 ± 2.25	8.9 ± 1.65	0.014 *^b^
TLC	46.2 ± 89.8	12.5 ± 23.7	0.001 *^b^
Lymphocytes			
Normal	20 (38.1)	11 (53.2)	0.464 ^a^
Lymphopenia	25 (38.1)	22 (25.5)	
Lymphocytosis	19 (30.2)	10 (21.3)	
Neutrophils			
Normal	19 (29.7)	7 (16.3)	0.284 ^a^
Neutropenia	26 (40.6)	21 (48.8)	
Neutrophilia	19 (29.7)	15 (34.9)	0.012 *^a^
Thrombocytes			
Normal	31 (48.4)	9 (20.9)	
Thrombocytopenia	27 (42.2)	30 (69.8)	
Thrombocytosis	6 (9.4)	4 (9.3)	
PT (seconds)	14.6 ± 2.90	14.9 ± 3.09	0.225 ^b^
PC (%)	75.5 ± 17.0	72.2 ± 16.8	0.801 ^b^
INR	1.21 ± 0.22	1.25 ± 0.25	0.254 ^b^
Creatinine (mg/dL)	0.74 ± 0.33	0.94 ± 0.68	0.002 *^b^
ALT (U/L)	40.3 ± 64.5	34.6 ± 50.1	0.546 ^b^
AST (U/L)	39.3 ± 44.3	51.5 ± 67.9	0.054 ^b^
CRP	110.9 ± 119.1	153.0 ± 15.1	0.594 ^b^
LDH (U/L)	730.1 ± 845.4	975.8 ± 904.43	0.184 ^b^

Both ^a^ Chi square and ^b^ Student *t* test were used for statistical analysis. The mean differences and Std. Deviation, (*) refers to 2-tailed Sig. ICU: Intensive Care Unit. Hb: Hemoglobin. LC: Leukocyte count. PT: Prothrombin time. PC: Prothrombin concentration. INR: International normalized ratio. ALT: Alanine transaminase. AST: Aspartate transaminase. CRP: C-reactive protein. LDH: Lactate dehydrogenase.

**Table 5:** Multivariate analysis of variance (MANOVA) on clinical variables.

Source	Dependent Variable	Type III Sum of Squares	df	Mean Square	F	Sig.
Corrected model	ICU stay days	551.917	5	110.383	3.989	0.023
	TLC	73,518.166	5	14,703.633	6.161	0.005
	Neutrophil	10.194	5	2.039	4.382	0.017
	Hb	61.588	5	12.318	3.996	0.023
Intercept	Age	52.009	1	52.009	22.833	0.000
	Sex	17.763	1	17.763	71.053	0.000
	ICU stay days	993.265	1	993.265	35.892	0.000
	Mechanical Ventilation	0.634	1	0.634	4.803	0.049
	pH	619.501	1	619.501	1.090 × 10^5^	0.000
	pCo_2_	14,590.580	1	14,590.580	469.062	0.000
	pO_2_	43,253.003	1	43,253.003	26.996	0.000
	HCO_3_	5170.527	1	5170.527	599.506	0.000
	TLC	14,086.373	1	14,086.373	5.902	0.032
	Lymphocytes	15.107	1	15.107	21.121	0.001
	Thrombocytes	17.763	1	17.763	30.451	0.000
	Neutrophils	6.634	1	6.634	14.258	0.003
	Hb	1062.926	1	1062.926	344.842	0.000
	Pt	2143.701	1	2143.701	605.696	0.000
	PC	67,005.179	1	67,005.179	295.289	0.000
	INR	15.455	1	15.455	551.878	0.000
	CRP	145,320.421	1	145,320.421	7.459	0.018
	LDH	1.055 × 10^7^	1	1.055 × 10^7^	9.829	0.009
	AST	34,268.002	1	34,268.002	6.168	0.029
	Creatinine	6.338	1	6.338	34.558	0.000
Fatality	ICU stay days	418.792	1	418.792	15.133	0.002
Type of tumors	TLC	26,090.751	2	13,045.376	5.466	0.021
	Neutrophil	10.026	2	5.013	10.774	0.002
	Hb	33.842	2	16.921	5.490	0.020
Fatality vs. type of tumor	TLC	21,695.325	2	10,847.663	4.545	0.034

## Data Availability

Dataset available on request from the authors. The raw data supporting the conclusions of this article will be made available by the authors on request.
